# The Conformational Stability and Biophysical Properties of the Eukaryotic Thioredoxins of *Pisum Sativum* Are Not Family-Conserved

**DOI:** 10.1371/journal.pone.0017068

**Published:** 2011-02-22

**Authors:** David Aguado-Llera, Ana Isabel Martínez-Gómez, Jesús Prieto, Marco Marenchino, José Angel Traverso, Javier Gómez, Ana Chueca, José L. Neira

**Affiliations:** 1 Instituto de Biología Molecular y Celular, Universidad Miguel Hernández, Elche (Alicante), Spain; 2 Departamento de Química-Física, Bioquímica y Química Inorgánica, Universidad de Almería, Almería, Spain; 3 Departamento de Biología Estructural y Biocomputación, Centro Nacional de Investigaciones Oncológicas (CNIO), Madrid, Spain; 4 Departamento de Bioquímica, Biología Celular y Molecular de Plantas, Estación Experimental Zaidin, Consejo Superior de Investigaciones Científicas (CSIC), Granada, Spain; 5 Biocomputation and Complex Systems Physics Institute, Zaragoza, Spain; National Institute for Medical Research, Medical Research Council, London, United Kingdom

## Abstract

Thioredoxins (TRXs) are ubiquitous proteins involved in redox processes. About forty genes encode TRX or TRX-related proteins in plants, grouped in different families according to their subcellular localization. For instance, the *h-*type TRXs are located in cytoplasm or mitochondria, whereas *f*-type TRXs have a plastidial origin, although both types of proteins have an eukaryotic origin as opposed to other TRXs. Herein, we study the conformational and the biophysical features of TRX*h*1, TRX*h*2 and TRX*f* from *Pisum sativum*. The modelled structures of the three proteins show the well-known TRX fold. While sharing similar pH-denaturations features, the chemical and thermal stabilities are different, being PsTRX*h*1 (*Pisum sativum* thioredoxin *h*1) the most stable isoform; moreover, the three proteins follow a three-state denaturation model, during the chemical-denaturations. These differences in the thermal- and chemical-denaturations result from changes, in a broad sense, of the several ASAs (accessible surface areas) of the proteins. Thus, although a strong relationship can be found between the primary amino acid sequence and the structure among TRXs, that between the residue sequence and the conformational stability and biophysical properties is not. We discuss how these differences in the biophysical properties of TRXs determine their unique functions in pea, and we show how residues involved in the biophysical features described (pH-titrations, dimerizations and chemical-denaturations) belong to regions involved in interaction with other proteins. Our results suggest that the sequence demands of protein-protein function are relatively rigid, with different protein-binding pockets (some in common) for each of the three proteins, but the demands of structure and conformational stability *per se* (as long as there is a maintained core), are less so.

## Introduction

Thioredoxins (TRXs) are small molecular weight proteins (∼14 kDa), which are present in all organisms from bacteria to mammals. They are heat-stable proteins with a characteristic folding – consisting of a four-stranded β-sheet, sandwiched by three α-helices: a βαβαβαββα- and redox activity, mediated by the conserved WC(G/P)PC motif [1]-[3]; this motif remains unaltered in all the TRX sequences reported to date, regardless of the sequence variation in the rest of the primary structure; the active site cysteines are oxidized, being the key to the ability of thioredoxins to reduce other proteins. TRXs are able to interact with several targets playing a crucial role in many cellular redox metabolism and regulation processes [2], [4], such as blood clotting, cell proliferation, insulin degradation, seed germination and repair of oxidative damage. As a consequence TRXs are associated with a number of human pathologies, such as cancer, cardiac disease or viral infections; furthermore, recently, TRXs have been used as potential therapeutic regulators of cell growth, apoptosis and inflammation [5].

Since they were first described by Reichard and co-workers [6], the number of TRX members identified in prokaryotes and eukaryotes has increased dramatically. Two families of TRXs can be distinguished based on their sequence. Family I includes proteins with a single TRX domain, whereas family II is composed of multiple TRX domains. At least twenty TRX-encoding genes belonging to family I have been reported in *Arabidopsis thaliana*, while in mammals there are only two, as in *E. coli* [7], [8]. Members of the family I in higher plants can be classified in six subgroups: TRX*f*, TRX*m*, TRX*h*, TRX*o*, TRX*x* and TRX*y* [9]. The TRX types *f*, *m*, *x* and *y* are present in plastids, type *h* is found in cytoplasm or mitochondria, and type *o* is localized in mitochondria [10]-[17]. The TRX types *m*, *x* and y are related to prokaryotic TRXs, while types *f*, *h* and *o* are related to eukaryotic organisms [18]-[20].

The *h*-type TRXs have been considered the most complex group of family I [19]. This type is divided in three subgroups, based on their primary sequence [16]: members of the first and second subgroup are kept in the reduced state by the thioredoxin reductase (NTR (NADPH-dependent thioredoxin reductase)) in a NADPH-(reduced species of the nicotinamide-adenine dinucleotide phosphate) dependent reaction, whereas the third subgroup is reduced by glutaredoxins, but not by NTR [9]. TRX*h* seems to play important roles during plant development, although its exact function remains unknown [9], [21]; interestingly enough, TRX*h* is able to decrease the allergic response to wheat proteins [22], and thus, it could be a promising therapeutic protein.

To date, eight TRX-encoding genes have been found in *Pisum sativum*, and four belong to the TRX*h* type [21], [23], [24]; furthermore, PsTRX*h*1 and PsTRX*h*2 (*Pisum sativum* thioredoxin *h*2) show different protein-binding properties [25], and little is known about the differences in the recognition of their substrates. On the other hand, although PsTRX*f* (*Pisum sativum* thioredoxin *f*) is located at a different cellular compartment, it is also specific of eukaryotic organisms. Then, in this work, we describe and compare the biophysical properties of the PsTRX*h*1, PsTRX*h*2 and PsTRX*f* to shed light on the differences observed in recognizing their protein targets, and in their physiological roles. Moreover, it has been shown that the redox enzymatic mechanism of eukaryotic-origin TRXs is different from that of bacterial-origin TRXs [26], showing a single electron transfer reaction; thus, it is interesting to elucidate whether that common enzymatic mechanism also yields to a common conformational stability and similar biophysical properties. Our results show that PsTRX*h*1, PsTRX*h*2 and PsTRX*f* have a similar pH-denaturation profile, whereas the thermal and chemical-denaturation profiles are different: PsTRX*h*2 follows a thermal three-state unfolding behaviour, and the PsTRX*h*1 is, on the other hand, the most stable. We can relate these differences in stability to the different amount of ASAs. We also discuss how the unique conformational features of each TRX isoform determine its different function and protein-protein recognition patterns. Thus, we show that the small differences observed in the pH denaturation profiles are related to the functions carried out by each PsTRX; furthermore, residues which seem to be responsible of a change in a particular biophysical feature (for instance, dimerization or chemical-denaturation), intervene in distinct binding regions to other proteins in each TRX.

## Materials and Methods

### Materials

Deuterium oxide (99% atom in ^2^H_2_O) was obtained from Apollo Scientific Ltd. (UK). Ultra-pure GdmCl (guanidinium hydrochloride) was from ICN Biomedicals Inc. (USA). The maltose-TEV (tobacco etch virus) protease was requested from the Harvard depository of clones (http://plasmid.med.harvard.edu/PLASMID/) and expressed and purified as described [27]. Standard suppliers were used for all other chemicals. Water was deionized and purified on a Millipore system.

Site directed mutagenesis at position Cys12 in PsTRX*h*1, and at position Cys56 in PsTRX*f* were carried out by using the Quick-Change-Site-Directed Mutagenesis kit (Stratagene, USA). Oligonucleotides containing the corresponding mutations were designed according to the manufacturer instructions, and their concentration was determined spectrophotometrically.

### Protein expression and purification

The cDNAs from PSTRX*f*, PsTRX*h*1 and PsTRX*h*2 [21] were subcloned using BamHI and HindIII restriction sites into a pETm11 plasmid (Novagen), and modified to yield a protease cleavable N-terminal hexa-hystidine (His_6_)-tag. The TEV cleavage sequence is formed by –ENLYFQG-, and the protease cleaves between the Q and G residues. Plasmid encoding (His_6_)-tagged proteins were transformed into *E. coli* BL21. Transformed cells were grown at 37°C of LB media (containing 50 µg/ml ampicillin) to OD_600_ ∼ 0.6–0.9. Protein expression was induced by addition of 1 mM IPTG. After addition of IPTG, the cells were incubated overnight at 37°C and harvested by centrifugation.

Proteins were purified by Ni^2+^-affinity chromatography in a pre-packed HiTrap affinity columns (GE Healthcare, Spain) according to the manufacturer's indications. Briefly, cell pellets were resuspended in 50 mM Tris (pH 8.0), 1% Triton X-100, 0.5 M NaCl, 1 mM β-mercaptoethanol, 10 mM imidazole (buffer A+1% Triton X-100) and lysed by sonication on ice. Insoluble cell debris was removed from the cell lysate by centrifugation. The supernatant was loaded onto a HiTrap affinity column, equilibrated with buffer A. The proteins were eluted with a linear gradient of buffer B (buffer A+1 M imidazole). Fractions containing the target protein were pooled together, concentrated, and loaded onto a Superdex 75 16/60 size-exclusion column (GE Healthcare) equilibrated with 25 mM phosphate (pH 7.0) plus 150 mM NaCl, on an AKTA FPLC (GE Healthcare) by following the absorbance at 280 nm. The (His_6_)-tag was cleaved off by using the *in-house* expressed and purified TEV. The concentration of pure proteins was determined by measuring the absorbance at 280 nm with the extinction coefficients as determined by amino acid sequence data [28]. The number of residues and other biophysical data for each TRX are shown in Table 1.

**Table 1:** Biophysical parameters of the three PsTRXs.

Biophysical parameter	PsTRX*h*1	PsTRX*h*2	PsTRX*f*
Molecular weight (kDa)	13.1	13.0	12.1
Theoretical *R*_S_ (Å) a	19±4	19±4	18±4
Theoretical r_0_ (Å) b	15.6	15.5	15.2
Number of residues	120	118	109
Number of fluorescent residues (W, Y)	3, 0	3, 0	2, 2
Theoretical pI^c^	5.67	4.62	7.82

a Determined according to the equation described by Dobson and co-workers [49].

b Determined from 
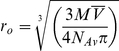
 [48].

c Determined from their sequences by the ExpasY web-tools: http://www.expasy.ch/tools/protparam.

### Analytical ultracentrifugation

Sedimentation equilibrium experiments were performed at 25°C in an Optima XL-A (Beckman-Coulter Inc.) analytical ultracentrifuge at rotor speeds of 19000, 22000, and 29000 rpm in double-sector charcoal-filled Epon centerpiece. Absorbance data at 280 nm were acquired from 90 µl samples at the concentration of 150 µM in 50 mM Tris (pH 8.0). The data from all speeds and loading concentrations were analyzed globally using the program SEDPHAT (http://www.analyticalultracentrifugation.com/sedphat/).

Sedimentation velocity experiments were performed with a XL-A analytical ultracentrifuge (Beckman-Coulter Inc.). A total of 390 µl samples in 50 mM Tris (pH 8.0), at the concentration of 150 µM, were used in a standard 12 mm charcoal-filled Epon double-sector centrepiece equipped with sapphire windows, inserted in an An50 Ti eight-hole rotor. Absorbance data were acquired at rotor speed of 42000 rpm and at a temperature of 20°C. Data were modelled as a superposition of Lamm equation solutions with the SEDFIT software [29]. The sedimentation coefficient distribution, c(s), was calculated at a confidence level of p = 0.68. The experimental sedimentation values were determined by integration of the main peak of c(s) and corrected to standard conditions to get the corresponding s_20,w_ values with the SEDNTERP program [30]. Calculation of frictional coefficient ratio was performed with the SEDFIT program to obtain the c(M) distribution [29].

### NMR spectroscopy

Spectra were recorded on a Bruker Avance DRX-500 spectrometer, working at a ^1^H frequency of 500.13 MHz, and equipped with a z-axis gradient field probe. Experiments were acquired at 25°C. Samples were prepared by dissolving the lyophilized protein in a 9:1 H_2_O:^2^H_2_O solution, at a final protein concentration of 0.2 mM. The solution was centrifuged briefly to remove insoluble protein and then transferred to a 5-mm NMR tube. The pH of the sample was adjusted by small amounts of ^2^HCl and NaO^2^H to the final value of 5.8, since at this pH no evidence of the dimeric form in some TRXs was observed (see Results). Values of the pH reported represent apparent values of pH, without correction for isotope effects. The TSP (trimethyl-silil-propionate) was used as an external chemical shift reference.

Measurements of the *T*_2_ (transverse relaxation time) provide a convenient method to determine the molecular mass of a macromolecule, since the correlation time, τ_c_, is approximately equal to 1/(5*T*_2_) [31], with an inherent uncertainty of 10%. We measured the *T*_2_ for the three TRXs with the 1-1 echo sequence [32].

### Fluorescence

The fluorescence spectra were collected at 25°C on a Cary Varian spectrofluorimeter (Varian, USA), equipped with a Peltier thermoelectric temperature controller. A 1-cm pathlength quartz cell (Hellma) and protein sample concentrations of 2 µM were used in 100 mM of the corresponding buffer. The salts and acids used in buffer preparation were: pH 2.0–3.0, phosphoric acid; pH 3.0–4.0, formic acid; pH 4.0–5.5, acetic acid; pH 6.0–7.0, NaH_2_PO_4_; pH 7.5–9.0, Tris acid; pH 9.5–11.0, Na_2_CO_3_; pH 11.5–13.0, Na_3_PO_4_. The pH was measured with an ultra-thin Aldrich electrode in a Radiometer (Copenhagen, Denmark) pH-meter.

### (a) Intrinsic fluorescence

Protein samples were excited at 280 nm and 295 nm. The slit widths were 5 nm for the excitation and emission lights. Spectra were recorded between 300 and 400 nm. The signal was acquired for 1 s and the wavelength increment was set to 1 nm. Blank corrections were made in all spectra. No differences in the maxima wavelengths were observed for the three TRXs at the two excitation wavelengths (data not shown).

GdmCl titrations either followed by fluorescence or CD (circular dichroism) were carried out at different pHs; the proper amount of the denaturant from a 7 M stock solution was used and samples were left overnight to equilibrate at 4° C. Reversibility experiments were carried out by dissolving a stock solution of the corresponding TRXs in 7 M GdmCl. Each chemical denaturation was repeated three times with new samples.

### (b) Thermal denaturations

The excitation wavelengths were 280 and 295 nm, and fluorescence emission was collected at 315, 325, 340 and 350 nm. Slit widths were 5 nm for excitation and emission light. Thermal scans were acquired every 0.2°C with a heating rate of 60°C/h. Every thermal denaturation was repeated three times with new samples.

### (c) ANS (8-anilinonaphtalene-1-sulfonic acid)-binding

Excitation wavelength was set at 380 nm, and the emission fluorescence was measured from 400 to 600 nm. Stock solutions of ANS were prepared in water and diluted into the samples to yield a final 100 µM dye concentration. Dye concentrations were determined using an extinction coefficient of 8000 M^−1^ cm^−1^ at 372 nm. In all cases, blank solutions were subtracted from the corresponding spectra.

### Circular dichroism

Circular dichroism spectra were collected on a Jasco J810 (Japan) spectropolarimeter equipped with a Peltier thermoelectric temperature controller. The instrument was periodically calibrated with (+) 10-camphorsulphonic acid.

### (a) Steady-state measurements

Far UV-CD spectra at a protein concentration of 10 µM were recorded at 25°C with a 0.1-cm pathlength cell, a scanning speed of 50 nm/min and a response time of 2 s. The spectra reported are the average of six scans, corrected by subtracting the corresponding spectra of the buffer. All proteins were dissolved in 10 mM of the suitable buffer to cover a pH range from 2.0 to 13.0. Samples were left to incubate overnight at 4° C.

### (b) Thermal denaturations

Experiments were performed using a constant heating rate of 60°C/h following the ellipticity at 222 nm from 25 to 95°C in 0.1-cm cells, with a total protein concentration of 10 µM, a response time of 8 s and a data pitch of 0.2°C. Experiments were repeated three times with new samples. The reversibility of the thermal transitions was confirmed by comparing the CD spectra acquired before and after thermal denaturation. Instrument drifting was excluded by comparing the buffer scans before and after the thermal experiments; no difference was observed between the scans.

### Analysis of the chemical- and thermal-denaturation curves, and free-energy determination

The average emission intensity, <λ>, in fluorescence spectra was calculated as [33]: 
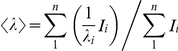
, where *I*_i_ is the fluorescence intensity measured at a λ_i_ wavelength.

Chemical-denaturation curves were analysed using a two-state model for the native/unfolded equilibrium, according to the linear extrapolation model: Δ*G* = *m*([U]_50%_ - [U]), where Δ*G* is the free energy of denaturation, [U] is the denaturant concentration, and [U]_50%_ is the denaturant concentration at the midpoint of the transition. The denaturation data from far-UV CD and fluorescence were fitted to the two-state equation [34]: 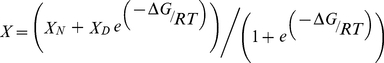
(1)where X*_N_* and X*_D_* are the corresponding physical properties (ellipticity at 222 nm, fluorescence intensity or <λ>) of the folded (N) and unfolded states (U), respectively, for which a linear relationship with the denaturant (i.e., X*_N_* = α*_N_*+β*_N_*[U] and X*_D_* = α*_D_*+β*_D_*[U]) is admitted; *R* is the gas constant and *T* is the absolute temperature in K. For PsTRX*h*2, the [U]_50%_ of both denaturation curves (followed by CD and fluorescence) are well-resolved (see Results) and the above equation can be applied, but we also carried out a three-state analysis of the GdmCl-denaturation data leading to a result similar to the two-state model, within the experimental error (data not shown) (see [35] for a discussion).

In thermal-denaturations, the Δ*G* was given by [36]:
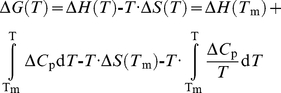
(2)where Δ*S*(*T*_m_) and Δ*H*(*T*_m_) are the entropy and enthalpy of unfolding at the thermal denaturation midpoint, *T*_m_, respectively, and Δ*C*_p_ is the heat capacity of the transition The shape of equation (1) (with exponential terms above and below the rate) does not impose restrictions on the value of the Δ*C*_p_; thus, fitting of the experimental data (see Results) and the exact determination of the *T*_m_, does not rely on a pre-fixed value of the heat capacity [37]. In fact, we did not find any difference in the final fitted value of *T*_m_ when Δ*C*_p_ was either fixed (to 1200 cal mol^−1^ K^−1^, a usual standard value [36]) or calculated during the iterative procedure. Fitting by non-linear least-squares analysis to the above equations was carried out by using Kaleidagraph (Abelbeck software) on a PC computer.

### Differential Scanning Calorimetry (DSC)

DSC experiments were carried out with a VP-DSC calorimeter (MicroCal, Northampton, MA). Protein solutions were prepared by exhaustive dialysis at 5°C, against the working buffer (10 mM MES (pH 7.3), 200 mM NaCl). To minimize the amount of gas dissolved in the solutions, all the samples were degassed under vacuum for 10 min at room temperature with gentle stirring before being loaded into the calorimetric cells. Samples were heated at a constant scan rate of 1.0°C/min (60°C/h) and held under an extra pressure of 2 bars (28 psi) to avoid bubble formation and evaporation at high temperatures. Several buffer-buffer scans were performed to ensure proper instrument equilibration.

To test the reversibility of the thermal denaturation, protein solutions were cooled *in situ* to 20°C for 30 min immediately after the first scan was completed (usually, ranging from 20 to 80°C), and rescanned under the same experimental conditions. To correct for small mismatches between the two cells, an instrumental baseline (i.e., buffer-buffer baseline) was subtracted from the protein endotherm before data analysis. All traces were dynamically corrected to account for the time-delayed response of the detector to the heat event that evolved within the calorimetric cell. After normalization to concentration, a chemical baseline calculated from the progress of the unfolding transition was subtracted. Fitting was carried out by using the Origin 7.0 package supplied with the instrument.

### Size exclusion chromatograph (SEC)

Analytical gel filtration experiments were performed in a Superdex 75 HR 16/60 column (GE Healthcare) at 1 ml/min and at 25°C on an AKTA FPLC system, following absorbance at 280 nm. A volume of 100 µl at a concentration of 24 µM was loaded into the column after equilibration with the corresponding buffers (at 50 mM), at the suitable pH, and with 150 mM NaCl. The reported elution volumes are the average of three measurements. Experiments at different GdmCl concentrations were carried out a pH 7.3.

The column was calibrated using the gel filtration low-molecular-weight calibration kit (GE Healthcare). The standards and their corresponding Stokes radii were: ribonuclease A (16.4 Å); chymotrypsinogen (20.9 Å); ovoalbumin (30.5 Å), and bovine serum albumin (35.5 Å) [38]. The reported elution volumes are the average of three measurements.

The elution of a macromolecule in gel filtration experiments is usually given by the partition coefficient, which is defined as the fraction of solvent volume within the gel matrix accessible to the macromolecule [39]. The average weight partition coefficients (σ) of protein standards and PsTRXs were calculated by:
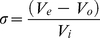
(3)where *V*_e_ is the elution volume of the protein, and, *V*_o_ and *V*_i_ are the void and internal volumes of the column, with values of 7.88±0.06 ml and 18.72±0.03 ml, respectively. The *V*_o_ and *V*_i_ volumes were determined, respectively, by using Blue dextran (5 mg/ml, in 10 mM phosphate buffer, pH = 7.0, containing 150 mM NaCl) and riboflavin (0.5 mg/ml, in the same buffer), by averaging four measurements for each compound.

The partition coefficients were determined for the molecular-size standards and they were transformed by using the inverse error function complement of σ, (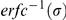), yielding a linear relationship with the molecular Stokes radius, *R*_s_ [39]:
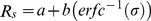
(4)where *a* and *b* are the calibration constants for the column.

Fitting of the calculated 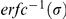 for the protein standards to equation (4) by linear least-squares analysis was carried out on Kaleidagraph (Abelbeck software) on a PC computer. The *R*_s_ of any macromolecule can be determined, when the calibration parameters, *a* and *b*, are obtained.

### Bioinformatics

Protein sequence alignment was carried out by using the program UTOPIA (user-friendly tools for operating informatic applications) CINEMA (colour interactive editor for multiple alignments) v 1.4.5 (http://utopia.cs.man.ac.uk/utopia/) [40].

Modelling was carried out by homology at the EsyPred3D Web Server 1.0 [41] using as template the structure of the TRX*h*1 from *Arabidopsis thaliana* (AtTRX*h*1: PDB entry 1XFL) which shares 70% and 64% similarity with PsTRX*h*1 and PsTRX*h*2, respectively; the structure of the TRX*f* from spinach (1FAA) was used for modelling the structure of PsTRX*f* (77% of sequence identity) [42]. The models contained residues 4 to 116 (94% coverage), 2 to 113 (95% coverage), 3 to 105 (96%) with 96%, 93.1% and 95% of residues in the most favoured regions of the Ramachandran plot for PsTRX*h*1 and PsTRX*h*2 and PsTRX*f*, respectively. The overall quality factors [43] were 94.231, 88.462 and 92.435 for TRX*h*1, TRX*h*2 and TRX*f* respectively, and 100%, 82.3% and 96.3% of the residues with an averaged 3D-1D score [44] larger than 0.2 for the three proteins. The structures modelled for the three PsTRXs are similar, and they show the typical TRX fold; the only difference is a smaller β-sheet, and a shorter α-helix at the N terminus in PsTRX*f*, which is absent in the other two TRXs.

We used the FPOCKET webserver (http://bioserv.rpbs.univ-paris-diderot.fr/fpocket/) to predict the possible binding pockets in the three modelled structures [45], [46]. The VADAR web-server was used to predict the structural parameters of the modelled structures [47] (http://redpoll.pharmacy.ualberta.ca/vadar/).

## Results

### PsTRXh1, PsTRXh2 and PsTRXf are monomeric proteins

The oligomerization state of the PsTRXs was determined comparing: (1) the estimated molecular weights from *T*_2_-relaxation time measurements; (2) the calculated *R*_S_ by SEC; and, (3) the protein species distribution obtained by AUC (analytical ultracentrifugation).

**Figure 1:**
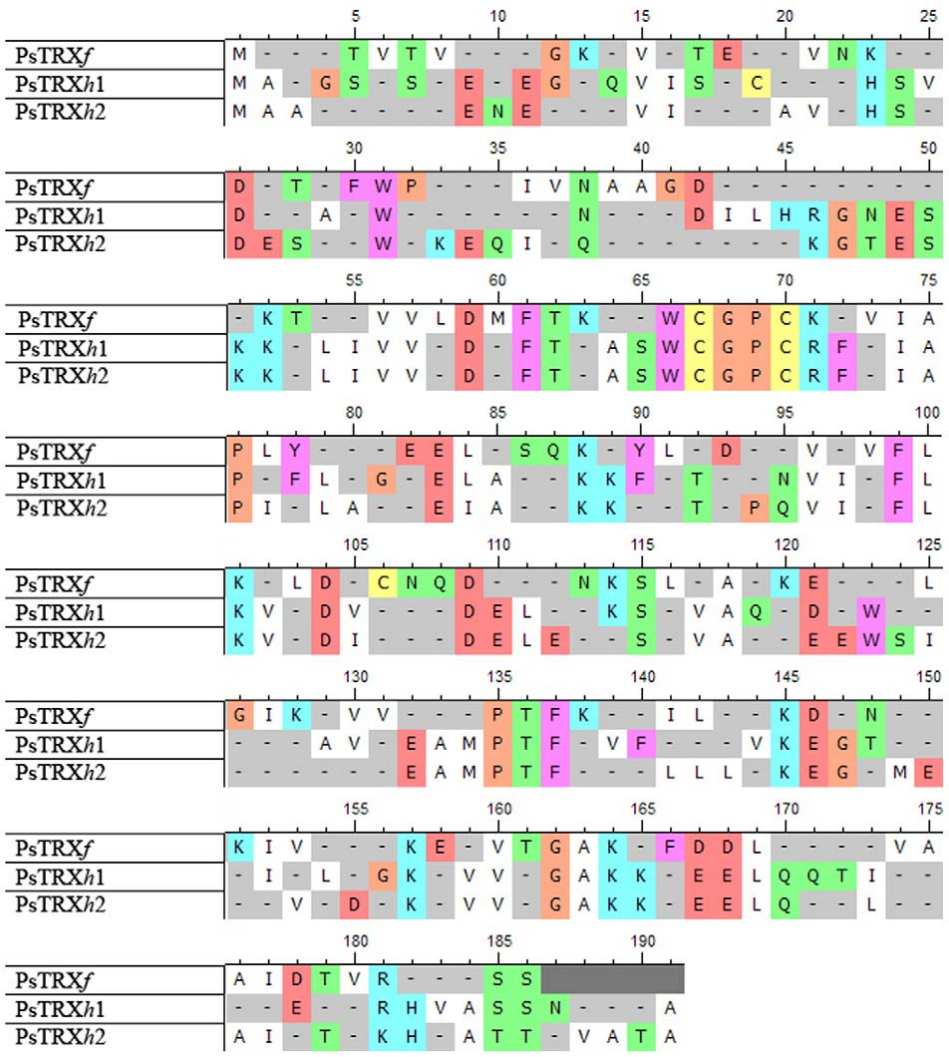
Sequence alignment of the three PsTRXs. The colour code indicates the different types of amino acids. Alignment and colour coding was carried out with the UTOPIA-CINEMA software. Metionine is not taken into account in the numbering of the sequences. The numbering of PsTRX*f* (first row) starts from the second valine in the sequence shown; the additional TVT sequence at is N terminus comes from cloning.

The *T*_2_-relaxation times of the most down-field shifted signals of the ^1^H-1D-NMR spectra of the PsTRXs are in Table 2. The estimated molecular weights are similar to those calculated from the corresponding sequences (Fig. 1, Table 1).

**Table 2:** Hydrodynamic properties of PsTRXs^a^.

Technique	Biophysical parameter	PsTRX*h*1	PsTRX*h*2	PsTRX*f*
NMR	*T*_2_-relaxation time (ms)	30.0	30.5	28.7
	τ_c_ (correlation time) (ns)	6.7	6.5	6.9
	Molecular weight (kDa)	13.4	13.1	13.7
SEC	*V*_e_ (ml)^b^	13.3±0.1	12.8±0.1	13.5±0.1
	Experimental *R*_S_ (Å)^c^	14±2	16±2	14±2
AUC^d^	Equilibrium sedimentation	13.8	13.1	11.6
	Sedimentation velocity	14.2	12.8	13.3

a Measurements were carried out at 25°C, pH 7.0.

b *V*_e_ is the elution volume of the main (monomeric) peak for PsTRX*h*1 and PsTRX*f*. The error is the standard deviation of three measurements.

c The calibration equation for the column is: *R*_S_ = 1.69 (±1.79)+26.7 (±1.56) erfc^-1^ (σ).

d The reported values are the molecular weights determined by both techniques.

The *V*_e_ together with the *R*_S_ are shown in Table 2. The elution profile of PsTRX*h*1 at pH≥7.0 consisted of two peaks at 12.8 and 13.3 ml; the peak at 13.3 ml corresponded to the expected monomeric species. We thought that the peak at 12.8 ml corresponded to the dimeric species of PsTRX*h*1 formed by a -S-S- bond mediated by the cysteine located outside the CXXC motif (Fig. 1): Cys12. To confirm this hypothesis, we added 100 µM DTT to the elution buffer, and the peak at 12.8 ml disappeared. Moreover, an additional control came from the expression of the PsTRX*h*1C12S mutant, which eluted only as a monomer at *V*_e_ = 13.4 ml (data not shown). Interestingly, PsTRX*f* showed an elution profile similar to PsTRX*h*1 at pH≥7.0, with two peaks at 11.43 and 13.5 ml. Similarly, the presence of DTT in the elution buffer, and the production of the corresponding PsTRX*f*C56S mutant confirmed the dimeric nature of the peak at 11.43 ml. Moreover, from the intensity ratios of the first and the second peak, we can also conclude that PsTRX*f* had a smaller tendency to self-associate than PsTRX*h*1.

The theoretical value of the Stokes radius, r_0_, for a spherical molecule can be determined by [48]: 
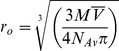
, where *M* is the molecular mass of the protein; 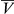
is the partial specific volume of the protein (we have taken for the three TRXs the average value, 0.73 cm^3^ g^−1^), and *N*_Av_ is Avogadro's number (Table 2). Dobson and co-workers have suggested that the *R*_S_ of a folded protein is given by: *R*_S_ = (4.75±1.11) *N*_u_^0.29^ [49], where *N*_u_ is the number of residues; this equation leads to the theoretical *R*_S_ values shown in Table 1. As observed, there is a good agreement between the theoretical and experimental hydrodynamic parameters, suggesting a roughly spherical shape for the three proteins.

**Figure 2:**
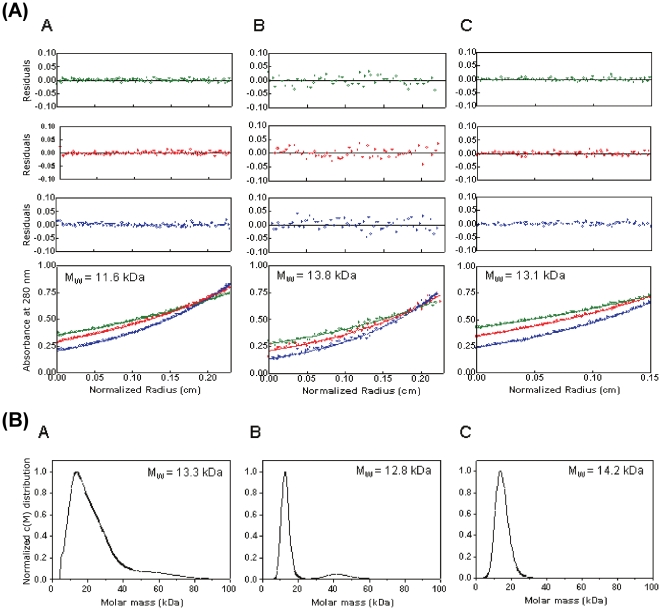
Analytical ultracentrifugation experiments. (A) Equilibrium sedimentation data. Samples of PsTRX*f* (A), PsTRX*h*1 (B), and PsTRX*h*2 (C) were centrifuged at 20°C at 19000 (green), 22000 (red) and 29000 (blue) rpm. The data from all speeds and loading concentrations were analyzed globally using the program SEDPHAT. Fits to a single species model are shown as solid lines and the corresponding residuals are shown. M_W_ is the molecular weight derived from the global fitting. (B) Sedimentation velocity data. Samples of PsTRX*f* (A, left column), PsTRX*h*1 (B, middle column), and PsTRX*h*2 (C, right column) were centrifuged at 42000 rpm at 20°C.

Since SEC measurements cannot distinguish contributions of mass and shape to molecular diffusion, and the *T*_2_-relaxation measurements only provide estimates, we decided to use AUC to determine directly the molecular weight of the three proteins [29]. The monomeric nature of PsTRXs was supported by AUC: sedimentation equilibrium measurements performed under the same solution conditions of SEC indicated that TRXs are monomeric (Fig. 2A). In sedimentation velocity experiments, PsTRX*h*2 sedimented with a molecular weight of 14.2 kDa (Figure 2B, right column), which is slightly larger than the expected theoretical value for a monomer. A similar behaviour was observed for PsTRX*f* and PsTRX*h*1, with estimated molecular weights of 13.3 and 12.8 kDa, respectively (Fig. 2B, left and middle columns). The sedimentation velocity profiles of PsTRX*h*1 and PsTRX*f* showed an asymmetric distribution (which is more evident in PsTRX*f*, Fig. 2B, left column), or alternatively, peaks at higher molecular weights (as in PsTRX*h*1) suggesting the presence of higher-order oligomeric species. Furthermore, PsTRX*f* had the highest difference in the determined molecular weight between the equilibrium and velocity measurements (Table 2).

We conclude that the three PsTRXs are mainly monomeric proteins over a wide pH range, and that the dimerizations of PsTRX*h*1 and PsTRX*f* at physiological pH result from the formation of an intermolecular disulphide bond through the non-active-site cysteines: Cys12 and Cys56, respectively.

### PsTRXh1, PsTRXh2 and PsTRXf show acidic denaturations with a molten-globule-like species

Next, we determined the biophysical features of the TRXs at different pHs by using four different techniques: intrinsic- and ANS-fluorescence, far-UV (ultraviolet) CD and SEC. The intrinsic fluorescence provides information on the tertiary structure around tryptophan and tyrosine residues [50]. The ANS-fluorescence is used to monitor the changes in solvent-exposed hydrophobic surface: ANS binds to spatially close solvent-exposed hydrophobic patches [51], shifting the fluorescence maxima from 520 (isolated ANS) to 480 nm (ANS-bound to a polypeptide hydrophobic patch). The far-UV CD allows us to monitor the secondary structure, and in some proteins, the structure around the aromatic residues [52]-[54]. And SEC provides information about the compactness of the protein [55].

The fluorescence spectra of PsTRX*h*1 and PsTRX*h*2 have been described previously [25]; briefly, both proteins show spectra dominated by the contribution of the three tryptophans (Table 1), which are partially-solvent exposed. The fluorescence spectrum of PsTRX*f* also shows the dominant contribution of a tryptophan, with a maximum wavelength close to 345 nm, suggesting a partial solvent-exposure of at least one of the two indole moieties. The far-UV CD spectra of PsTRX*h*1 and PsTRX*h*2 have also been described [25]; briefly, the spectrum of PsTRX*h*1 showed a minimum at 228 nm, which could be attributed to tryptophan residues [52], [53], [54], [56]; the far-UV spectrum of PsTRX*h*2 was less intense than that of PsTRX*h*1, with a small minimum at 222 nm, characteristic of helical proteins. The far-UV CD spectrum of PsTRX*f* also had an intense minimum at 228 nm, with a general shape similar to that of PsTRX*h*1.

#### (a) PsTRXh1

**Figure 3:**
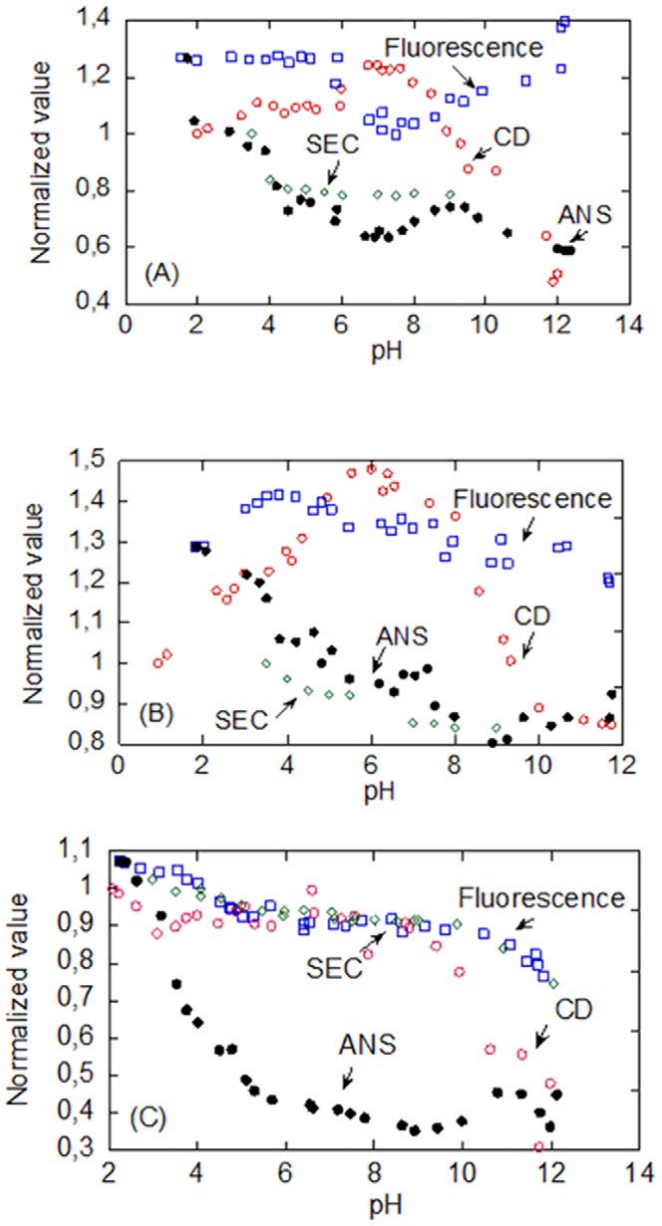
pH-denaturation behaviour. The pH behaviour of PsTRX*h*1 (A), PsTRX*h*2 (B), and PsTRX*f* (C) was monitored by CD (red blank circles); the <λ> of the intrinsic fluorescence (blue blank squares) by excitation at 280 nm (the same behaviour was observed by excitation at 295 nm (data not shown)); the <λ> of the ANS (black filled circles); and SEC (green blank diamonds). To allow for an easy comparison among the four biophysical techniques, all the values for each biophysical technique were normalized to the experimental data measured at the lowest pH in that particular technique. Experiments were acquired at 25°C. In the SEC experiments only the *V*_e_ of the peak corresponding to the monomeric species of PsTRX*h*1 and PsTRX*f* is shown.

The changes in the secondary structure of PsTRX*h*1, followed by CD, showed a bell-like shape tendency (Fig. 3A, red blank circles). There was an acidic titration (which was also monitored by ANS-fluorescence and SEC, see below) that should be due to titration of an Asp and/or Glu residues [57]; however, due to the absence of an acidic baseline, we could not determine reliably its p*K*_a_ (Fig. 3A). This acidic transition led to a reduction of the secondary structure as shown by the decrease (in absolute value) of the ellipticity of the far-UV CD, but there was residual ellipticty at the lowest pH. The midpoint of the transition at basic pHs, which must be associated at changes in the titration of the arginine and/or lysine residues [57], could not be determined either; this titration decreases the percentage of secondary structure (as shown by CD).

Although the fluorescence intensity at any wavelength follows a bell-shaped pH tendency (data not shown), the <λ> showed a titration midpoint of 6.3±0.3 (the average of four measurements) (Fig. 3A, blue blank squares) either by excitation at 280 or 295 nm; these results suggest that <λ> is more sensible to any change in the fluorescence spectrum than fluorescence intensity at a particular wavelength [33]. This transition is the same monitored by CD (Fig. 3A, red blank circles), suggesting that the conformational changes around the fluorescent residues could be either affecting the secondary structure of the protein, or alternatively, changes in aromatic residues are being also monitored by the far-UV CD, as described in other proteins [54], [58]. It could be thought that this titration is due to Cys12, since at this pH we observed two peaks in SEC (see above); however, titration pH-experiments with the mutant PsTRX*h*1C12S showed the same behaviour as the wild-type protein (data not shown). Then, since the sole residue left titrating at this pH is a histidine [57], and as PsTRX*h*1 has three histidine residues (namely, His13, His23 and His111), we suggest that any of them is responsible for that titration.

The ANS-fluorescence was also pH-dependent. At low pHs, the <λ> was larger, and it was reduced as the pH was increased (Fig. 3A, black filled circles). Furthermore, there was a small “bump” between pH 7.0 to 11.0, that leads to an estimation of two p*K*_a_s 8.1±0.1 and 10.4±0.2; these two transitions (which were also observed by following the changes in the intensity at any wavelength) must be associated with arginine and/or lysine residues [57]; the two transitions were also observed in the ANS-titration of PsTRX*h*1C12S mutant (data not shown).

The protein eluted at larger *V*_e_ at acidic pHs, probably due to protein-column interactions (Fig. 3A, green blank diamonds), and it eluted at 13.0 ml from pH 6.0 to 9.0. The *V*_e_ of the dimeric species did not show variations in the *V*_e_ between pH 7.0 and 13.0 (data not shown).

#### (b) PsTRXh2

As in PsTRX*h*1, intrinsic fluorescence, ANS-fluorescence and SEC indicate the presence of an acidic titration in PsTRX*h*2. The <λ> showed a titration at low and high pHs, whose p*K*_a_s could not be determined due to the absence of the acidic and basic baselines, respectively (Fig. 3B, blue blank squares) (a similar behaviour was observed by following the fluorescence intensities at any wavelength, data not shown). The <λ> of the ANS-fluorescence showed a titration at acidic pHs, whose p*K*_a_ could not be determined (Fig. 3B, black filled circles). The ellipticity at 222 nm had a V-shape with an acidic (p*K*_a_ = 5.1±0.1) and basic titrations (p*K*_a_ = 8.6±0.1); that is, the maximum (in absolute value) of the ellipticity is attained in a narrow pH range (between pH 6.0 and 7.0) (Fig. 3B, red blank circles). In the SEC experiments, the protein eluted at larger *V*_e_ at low pHs, suggesting protein-column interactions; interestingly enough, between pH 5.5 and 7.0, where the maximum of the ellipticity was attained, the elution peaks were broadened beyond detection (Fig. 3B, green blank diamonds).

#### (c) PsTRXf

The PsTRX*f* showed a titration at low pH, which was monitored by three out of the four biophysical probes used (all but far-UV CD, Fig 3C, red blank circles). However, whereas ANS (Fig. 3C, black filled circles) and SEC (green blank diamonds) did not yield a reliable titration midpoint due to the absence of acidic baseline, the variation in the <λ> of the intrinsic fluorescence led to a p*K*_a_ of 4.31±0.06 (Fig. 3C, blue blank squares). Since the *V*_e_s in SEC at low pHs are larger than those at physiological ones, we suggest that there are protein-column interactions, as in the other PsTRXs. The fluorescence and CD also showed a transition at basic pHs, whose p*K*_a_ could not be determined due to the absence of baseline (Fig. 3C), and which could be associated to the titration of arginine, lysine and/or tyrosine residues [57]. This titration causes a decrease (in absolute value) in the ellipticity in the far-UV CD, thus indicating the loss of helical structure.

What can be learnt from the whole set of pH-denaturation results? We can conclude that the three PsTRXs show acidic and base denaturations. The acidic loss of secondary and tertiary structures, and compactness, and the burial of hydrophobic surface occur concomitantly in the three TRXs. Furthermore, the three PsTRXs show a partially folded species at low pH, with residual secondary structure (as shown by the far-UV CD experiments), but lack of tertiary structure (as suggested by the fluorescence spectra), and solvent-exposure of hydrophobic patches (as indicated by the ANS experiments). These features indicate the presence of a molten-globule species [59].

### PsTRXh1, PsTRXh2 and PsTRXf show different thermal denaturation behaviours and stabilities

From the above studies we can say at which pHs the proteins acquired a native-like conformation, but then, were these native-like conformations equally stable among the three proteins? To address such question, we carried out far-UV CD and fluorescence thermal-denaturations over a broad pH range and, in addition, DSC measurements at pH 7.3.

#### (a) PsTRXh1

We have previously carried out stability measurements at pH 5.9, followed by CD, in a His-tagged protein [25]. At that pH, the protein was extremely stable, and only in the presence of GdmCl, we could estimate a *T*_m_ of 89±1°C. In this work, we used far-UV CD and fluorescence thermal denaturations at pHs 2.75, 4.1, 7.3, 9.0 and 13.0, and DSC at pH 7.3. The far-UV CD experiments showed the beginning of a sigmoidal curve at temperatures higher than 77°C at the acidic pHs (Table 3); whereas at pHs 7.3 and 9.0, a clear reversible sigmoidal transition was observed, and at pH 13.0 no transition was detected, probably due to the basic denaturation observed (Fig. 3A). When thermal denaturation was followed by fluorescence, no transition was observed at any pH (Table 3); this is probably due to the fact that the thermo-quenching of the tryptophans obscures the sigmoidal transition [50]. Furthermore, we could not determine a reliable transition by DSC, since either in the presence of GdmCl or in its absence, the protein aggregated. This fact is probably due to the high temperatures and large protein concentrations (0.5 mg/ml) used during the experiments. Aggregation and irreversibility have also been observed during the heat induced unfolding of TRX*h* from *Clamydomonas reinhardtii* [60].

**Table 3:** The *T*_m_s in the TRXs (in°C) as measured by different biophysical techniques^a^.

	PsTRX*h*1			PsTRX*h*2			PsTRX*f*		
pH	Fluorescence	CD	DSC	Fluorescence	CD	DSC	Fluorescence	CD	DSC
2.75	Not observed	≥90		Not observed	Not observed		Not observed	59.8±0.3	
4.1	Not observed	≥90		Not observed	65.2±0.2		Not observed	75.3±0.5	
7.3	Not observed	77±1	Not observed	66.1±0.5	66.5±0.2	66 (*T*_max_)^b^	Not observed	71.2±0.5	68.5^c^
9.0	Not observed	73±1		64±2	66.3±0.2		Not observed	Not observed	
13	Not observed	Not observed		Not observed	Not observed		Not observed	Not observed	

a The *T*_m_s from fluorescence and CD techniques were determined by fitting to the two-state process (Equation (2)).

b Fitting to a three-state process.

c Fitting to a two-state process. The transition was not reversible.

#### (b) PsTRXh2

We have previously carried out stability measurements at pH 5.9 followed by CD in a His-tagged protein [25]; at that pH, the protein has a *T*_m_ = 75.9±0.3°C. In this work, we used far-UV CD and fluorescence (at the same pHs as in PsTRX*h*1) and DSC at pH 7.3 to monitor thermal unfolding. The results suggest that the stability was lower than that of PsTRX*h*1. At pHs 2.75 and 13.0 none of the techniques showed a transition; at pH 7.3 and 9.0, both techniques showed essentially the same *T*_m_ (Table 3), but the reaction was not fully reversible. The fact that fluorescence reports the thermal unfolding, suggests that although there are the same number of tryptophans at similar positions in PsTRX*h*1 and PsTRX*h*2 (Fig. 1), their environments must be slightly different.

**Figure 4:**
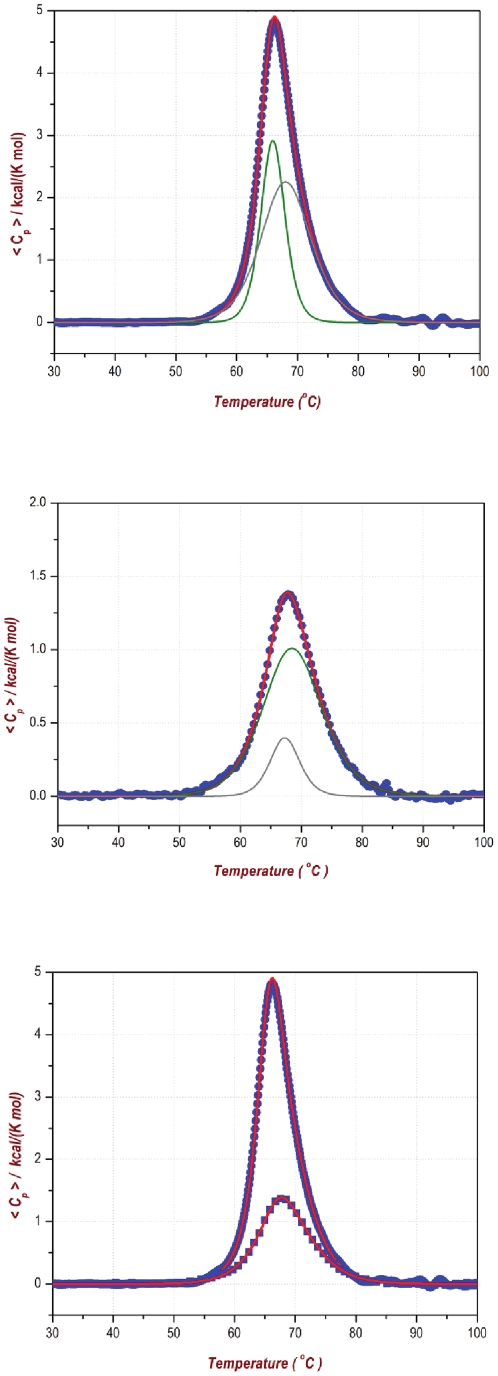
DSC of PsTRX*h*2. The top panel shows the calorimetric curve of the first scan with the analysis to a three-state model, indicating the two transitions (in green). The middle panel shows the calorimetric curve of the re-heating scan with a three-state model (in green), indicating the two transitions. The bottom panel shows both scans to allow for a comparison.

Conversely to what happens in PsTRX*h*1, a DSC thermogram could be obtained (Fig. 4). However, we could only fit the endotherm to a three-state transition, with a *T*_max_ = 66.3°C; furthermore, the transition was not fully reversible, since the *T*_max_ of the re-heating scan was 66.6°C, but the value of the Δ*H*_cal_ was four-times lower than that of the heating scan, suggesting that more of the 70% of the protein is unable to refold.

#### (c) PsTRXf

The fluorescence experiments did not show any sigmoidal transition probably due to the fact that the thermo-quenching of the two trypytophans obscures the sigmoidal transition (Table 3). Conversely, the far-UV CD only showed sigmoidal transitions at acidic and physiological pHs, probably due to the basic denaturation observed above pH 8.0 (Fig. 3C). The measured values of the *T*_m_s suggest that the protein stability is half-way between that of the PsTRX*h*1 (the highest) and PsTRX*h*2 (the lowest) (Table 3).

The DSC showed a non-reversible denaturation process (data not shown). There was a sole endotherm centered at 68.5°C, whose calorimetric enthalpy (Δ*H*_cal_ = 61.6 kcal/mol) is nearly identical to the van't Hoff one (Δ*H*_vH_ = 63.2 kcal/mol). Attempts to determine the Δ*C*_p_ from addition of small amounts of GdmCl failed, since the unfolding mechanism changed completely going from a two-state to a three-state system (data not shown).

To sum up, the three TRXs show a completely different behaviour during thermal denaturations: whereas in the more stable one (PsTRX*h*1), the DSC measurements did not yield reliable results due to aggregation, the less stable one (PsTRX*h*2) showed the presence of thermal intermediates.

### PsTRXh1, PsTRXh2 and PsTRXf show different chemical-denaturation behaviour

The above results suggest that the thermal denaturation behaviour is substantially different among the three proteins, but is it the same for the chemical denaturations? To answer this question we carried out GdmCl chemical-denaturations in the three proteins by using fluorescence, far-UV CD and SEC.

#### (a) PsTRXh1

**Figure 5:**
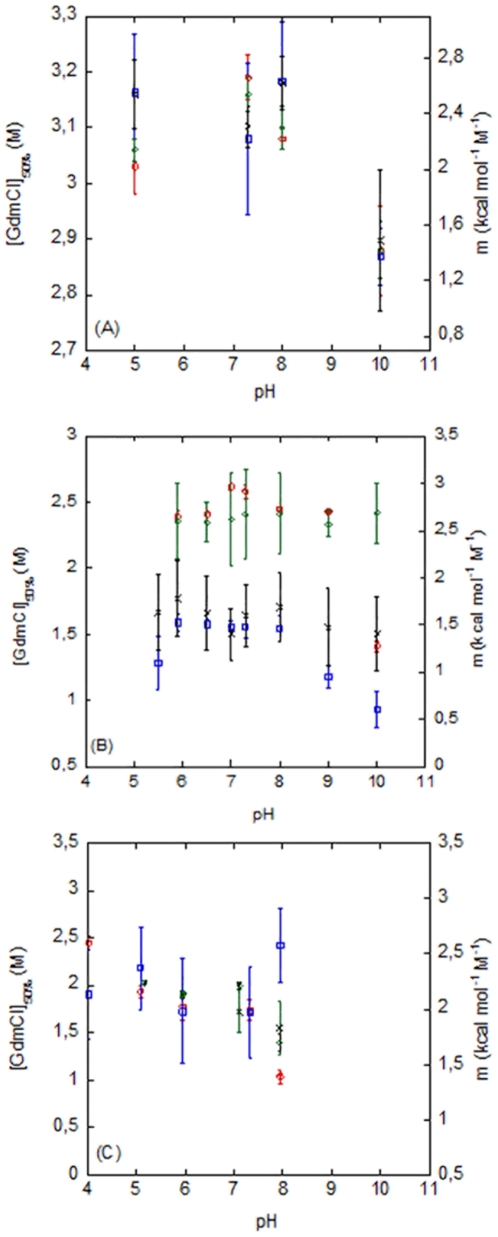
Chemical denaturations of the PsTRXs. The [GdmCl]_50%_ obtained by fluorescence (red blank circles), by CD (green blank diamonds) (left axis), and the *m*-values obtained by fluorescence (blue blank squares) and CD (black crosses) (right axis) are shown for PsTRX*h*1 (A); PsTRX*h*2 (B); and PsTRX*f* (C). Experiments were carried out at 25°C.

We have previously carried out chemical-denaturations of the His-tagged protein [25] by using fluorescence and CD at pH 5.9 (25°C), indicating that the protein is very stable. In this work, we go a step further and we tested the chemical-denaturation from pH 5.0 to 10.0. The [GdmCl]_50%_s determined by both techniques are the same within the experimental error (Fig. 5A), and the free energy, Δ*G*, is 7.3±0.5 kcal/mol (1 cal = 4.18 J). At pH 10.0, the stability of the protein did decrease, reaching a [GdmCl]_50%_ close to 2.8 M. The *m*-value in that pH range was constant, within the experimental error, and it averaged to 2.3±0.5 kcal mol^−1^ M^−1^ (by taking into account all the values measured by fluorescence and CD, except at pH 10.0). Comparison with those obtained in the His-tagged protein [25] indicates that the *m*- and the [GdmCl]_50%_-values are similar, and then the same Δ*G* is obtained in the tagged and cleaved protein, indicating that the presence of the tag did not affect the conformational stability of the protein. Then, we suggest that the observed differences in *T*_m_ between the tagged and the cleaved protein (see above) must be due to interactions between the tag and the protein at high temperatures.

We also followed the chemical denaturations by SEC; as the [GdmCl] increased, the *V*_e_ decreased suggesting a larger hydrodynamic radius due to unfolding of the protein. The chemical-denaturations followed by SEC had a [GdmCl]_50%_ of 2.76±0.02 M, with a steep *m*-value (i.e. a highly co-operative event), larger than that measured by using the spectroscopic techniques (Table 4). Then, the SEC results suggest that the compactness is lost at lower concentrations of denaturant than the secondary and tertiary structures, and thus, that the chemical-denaturation is not a two-state process. On the other hand, as the SEC measurements were carried out at pH 7.0, we were able to follow the chemical-denaturation of the dimeric species (data not shown): the [GdmCl]_50%_ of its transition (2.25±0.05 M) is similar to that of monomeric one (Table 4), and the *m*-value is less steep. Based on these results, we hypothesize that the dimeric species are two monomers tethered by the disulphide bridge, with a small number of interactions between them.

**Table 4:** The GdmCl chemical*-*denaturation parameters for the three PsTRXs at pH 7.0 and 25°C^a^.

	PsTRX*h*1		PsTRX*h*2		PsTRX*f*	
Technique	*m* (kcal mol^−1^ M^−1^)	[GdmCl]_50%_ (M)	*m* (kcal mol^−1^ M^−1^)	[GdmCl]_50%_ (M)	*m* (kcal mol^−1^ M^−1^)	[GdmCl]_50%_ (M)
Fluorescence	2.3±0.5	3.19±0.04	2.6±0.5	2.62±0.03	1.9±0.2	2.00±0.04
CD	2.3±0.2	3.16±0.03	1.4±0.3	1.56±0.04	1.9±0.4	1.8±0.1
SEC	11±3	2.76±0.01	6±1	1.80±0.02	Too broad to be determined^b^.	

a The errors are fitting errors to the two-state model. The chemical-denaturations were only fully reversible (either monitored by CD or fluorescence) in PsTRX*h*1; the chemical-denaturation of PsTRX*f* was partially reversible (see text for details), and that of PsTRX*h*2 was irreversible.

b The change occurred at ∼2 M GdmCl, going from *V*_e_ = 11.95 ml at [GdmCl] = 1.8 M to *V*_e_ = 10.29 ml at [GdmCl] = 2.0 M (data not shown).

#### (b) PsTRXh2

We have previously carried out chemical denaturations of a His-tagged PsTRX*h*2 by fluorescence and CD at pH 5.9 [25]. The results show that the protein is not as stable as PsTRX*h*1, and that the denaturation does not follow a two-state model, since two [GdmCl]_50%_ were obtained [25]. The same behaviour is observed at any pH (Fig. 5B), where the *m*- and [GdmCl]_1/2_-values from fluorescence are larger than those measured by far-UV CD. This result is surprising, since the tertiary structure (monitored by fluorescence) should be disrupted at smaller [GdmCl] than the secondary one (monitored by far-UV CD); such result can be rationalized, however, if we consider that fluorescence is monitoring the residual structure around a particular tryptophan residue (Table 1), and thus, it only reports a local denaturation event, as it has been described in other proteins [61]. Interestingly enough, no tyrosine residues are present in PsTRX*h*2, and PsTRX*h*1 and PsTRX*h*2 contain three indole moieties at similar positions in the primary structure (Fig. 1). Since the primary structure around the Trp15 (Trp18 in PsTRX*h*1) changes the most in both proteins (Fig. 1), we suggest that the differences in the chemical-denaturation observed in PsTRX*h*2, when compared to PsTRX*h*1, are due to the presence of residual structure around this indole moiety. This conclusion further supports our previous hypothesis (based on the thermal denaturation results, see above) that the trypotophan environments, despite their similar positions, are different in both proteins. The apparent [GdmCl]_50%_-values obtained by both techniques in PsTRX*h*2 remained constant in the pH range explored, and only at the extremes of pH (pH 5.5 and 10.0), it decreased (Fig. 5B). Finally, neither the transition followed by fluorescence nor that by CD were fully reversible; that is, the denaturation process of PsTRX*h*2 is irreversible in the concentration range from 1 to 10 µM.

Chemical-denaturation followed by SEC at pH 7.0 had a sigmoidal behaviour with a steep *m*-value and a [GdmCl]_50%_ = 1.80±0.02 M (Table 4), that is, closer (but not identical) to the value measured by CD (secondary structure). This result reinforces our previous hypothesis that fluorescence must be monitoring a local unfolding event involving some of the three tryptophans (see above), and it further pinpoints that the chemical denaturation does not follow a two-state model.

#### (c) PsTRXf

The conformational stability of PsTRX*f* was measured from pH 5.0 to 8.0. We followed the chemical-denaturation by using fluorescence, CD and SEC. The *m*- and [GdmCl]_50%_- values obtained by fluorescence and CD were identical, within the experimental error; furthermore, both parameters remained constant in the pH range explored, and only decreased at basic pHs (Fig. 5C). The chemical-denaturation followed by SEC did not yield reliable data since the transition was too broad (data not shown), with a [GdmCl]_50%_ ∼ 2.0 M and a large *m*-value (i.e., a highly cooperative event). As in the other two TRXs, this result suggests that the chemical-denaturation does not follow a two-state model; moreover, the [GdmCl]_50%_-values were not as high as those in PsTRX*h*1, but they were not as low as those measured in the far-UV CD for PsTRX*h*2 (Fig. 5B, Table 4).

When reversibility was tested by CD and fluorescence at pH 7.0, we observed that the [GdmCl]_50%_-value of the reversibility curve was the same (close to 2.0 M) but the *m*-value was slightly different to that measured in the unfolding curve (with a variation of the 15%); these findings mean that chemical-denaturation was not completely reversible.

To sum up, the chemical-denaturation of the three PsTRXs is different, but the three followed a three-state model, where the compactness (SEC) is lost at a first stage. Furthermore, as it occurs in the thermal denaturations, the conformational stability of PsTRX*f* is half-way between that of PsTRX*h*2 and PsTRX*h*1.

## Discussion

### The pH-unfolding of PsTRXs indicates the stabilization of a partially unfolded intermediate at low pH: evidence for molten-globule species

The acquisition of compactness (SEC), tertiary (fluorescence) and secondary structures (CD), and the burial of hydrophobic residues (ANS) occur simultaneously in the three PsTRXs; that is, the three TRXs from *Pisum sativum* behave similarly in their pH denaturation, showing the presence of a molten-globule-like protein. We do not know, however, whether these species are monomers; however, the fact that they elute at larger volumes than the corresponding folded TRX, suggests protein-column interactions, probably due to solvent-exposure of hydrophobic residues (which is further confirmed by ANS-binding experiments), and then, the possibility of aggregation to bury those solvent-exposed hydrophobic patches. The presence of this partially unfolded form in the three PsTRXs do not agree with the acidic denaturation observed in TRX of *E. coli*, where the secondary and tertiary structure of the protein does not change from pH 3.0 to 7.0 [62]; notwithstanding these facts, kinetic intermediates during the folding of *E. coli* TRX have been described by using several techniques [62]-[64]. The fact that the equilibrium folding intermediates in the TRX family have distinct structural features among the members (eukaryotic-origin *versus* bacterial-origin) does not agree with results found in the cytochrome c family, where the essential structural features of the folding intermediate are conserved [65]. Then, it is clear that more examples in other families must be described to allow for solid conclusions, but we suggest that the structural features of the folding intermediate are not conserved in the TRX family because TRXs are α+β proteins, a fold far more complex than the all α-helical fold of cytochrome.

The three proteins also show a basic denaturation, which must be associated with the titration of Lys or Arg residues [57], since PsTRX*h*1and PsTRX*h*2 do not have tyrosine residues (Table 1); this basic transition narrows in the latter proteins the pH range where both PsTRXs have a native-like conformation (Fig. 3). The fact that the pH range where PsTRX*f* has a native structure is larger than in the other two proteins can be explained on grounds of the different functions. PsTRX*f* is involved in the regulation of Calvin cycle proteins in plastids, and in this organelle the pH variation is larger than in cytoplasm (from pH 5.0 to 8.0 in the stromal chamber) [66]; thus, the larger number of lysine residues in the PsTRX*f*, and its basic nature (Table 1) allow a larger pH range where the protein can carry out its function, and, then, a different titration behaviour at basic pHs (when compared to other proteins), as monitored by fluorescence and far UV CD (Fig. 3).

On the other hand, neither the compactness nor the ANS-fluorescence is affected at higher pHs (Fig. 3); however, the ANS-fluorescence in PsTRX*h*1 showed a bell-shaped transition between 7.0 and 12.0, with p*K*_a_s at 8.0 and 10.0, which cannot be attributed to the Cys12, since the pH-titration of PsTRX*h*1C12S showed the same transitions (data not shown). Thus, we suggest that those transitions could be associated with a His, Lys and/or Arg residues [57]; PsTRX*f* has no histidine residues, PsTRX*h*2 has two histidine residues at similar positions than PsTRX*h*1 (Fig. 1), which also has a further additional histidine, His23. Moreover, His23 is adjacent to the hydrophobic patch Ile21-Leu22, which is not present in PsTRX*h*1 or in PsTRX*f* (Fig. 1), and it is followed by Arg24 (which is a Lys residue in PsTRX*h*1). Thus, we hypothesize that the change observed in the pH-titrations monitored by the ANS-fluorescence of PsTRX*h*1, is due to titration of His23, which motivates local rearrangement and solvent-exposure of residues.

Then, except for local structural rearrangements due to specific residues, the three PsTRXs show a concomitant acquisition of compactness, burial of hydrophobic residues, and acquisition of secondary and tertiary structures, with the presence of a molten-globule-like species at low pH.

### Dimerization of PsTRXh1 and PsTRXf through an additional cysteine: possible functional implications

**Figure 6:**
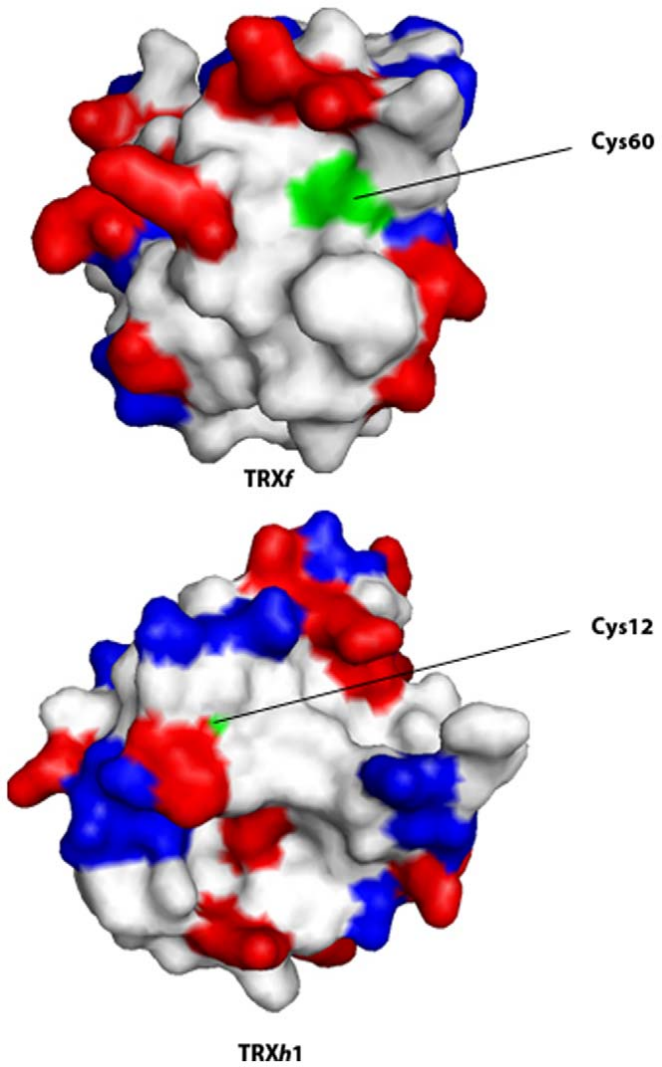
The additional Cys residues in PsTRX*h*1 and PsTRX*f*. The van der Waals surfaces of the modelled structures of both proteins are shown. The positievely charged residues are in red, and those negatively charged are in blue. The figures were produced with PyMOL [82].

It has been widely debated that TRXs have a tendency to dimerize [67]. The AUC and the *T*_2_-relaxation experiments show that the three PsTRXs are mainly monomeric at physiological pH, but there is evidence of a population of self-associated species in PsTRX*f* and in PsTRX*h*1 as detected by AUC (Fig. 2B). Furthermore, the SEC experiments in PsTRX*h*1 and PsTRX*f* suggest the presence of dimeric species, which involve disulphide formation (see Results), *via* two cysteines, not belonging to the active site, and which have a smaller p*K*_a_ (∼7.5), than that expected from models (∼8.5) [57], probably due to the fact that they are surrounded by a high number of positive residues (Cys12 in PsTRX*h*1) or are solvent-exposed (Cys60 in PsTRX*f*) (Fig. 6).

The Cys12 in PsTRX*h*1 is conserved in other *h*-type TRX, as in *Populus tremula* TRX*h*1, where dimeric forms have been also reported [68]; this region is surrounded by a positively charged patch (Fig. 6). There is no experimental evidence for the functional significance of the dimeric PsTRX*h*1 form, although we hypothesize that dimerization might be involved in protein activity regulation or protein-protein interactions. In humans, for instance, where a TRX1 isoform dimerizes through Cys73, the dimeric species is involved in detection of oxidative stress [69]. Moreover, Maeda and co-workers [70] have suggested that the dimerization of TRX*h*1 from *Hordeum vulgare* (HvTRX*h*1) (through a residue not belonging to the active site) is involved in substrate recognition. Since PsTRX*h*1 involves the same Cys as HvTRX*h*1, and the structural and sequence requirements are similar for both TRX*h*1s (data not shown), we hypothesize that the dimerization event in PsTRX*h*1 might be a distinct regulatory mechanism of protein-protein interactions; for instance, pull-down experiments at pH 7.5 with PsTRX*h*1 have shown, for the first time, a transcription factor able to bind a TRX molecule [25].

On the other hand, dimer formation of PsTRX*f* through Cys60, could be related to its well-known interaction with chloroplastic Fbase (fructose biphosphatase), where self-associated species of TRX have been shown to be required for proper binding [71]. This cysteine is highly solvent-exposed (Fig. 6), and it has been suggested to be involved in glutathionylation as a redox control mechanism, since glutathionylation reduces the ability of the TRX*f* to be reduced by FTR (ferredoxin-thioredoxin reductase) [72]. Finally, we hypothesize that the dimerization of TRX*f* might be protecting the FBase binding site, as it has been reported in other TRXs domains [73].

### The relationship between amino acid sequence and conformational stability in the PsTRXs is not robust

The molecular determinants of protein stability are very complex and not fully understood [74]. However, an increase in thermostability is generally caused by the additive effect of several and subtle changes, such as a slightly higher number of electrostatic interactions (salt bridges and hydrogen bonds, which, in turn, depend on the microenvironment surrounding a particular bond [75], an increase in the fractional polar surface, a decrease in the number of loops and turns, or the stabilization of α-helices [76].

The most stable of the three TRXs explored in this work is PsTRX*h*1, followed by PsTRX*f* and finally, the less stable one is PsTRX*h*2. It could be thought that the higher stability of PsTRX*h*1 can be due to the large amount of alkyl-chain hydrophobic residues (Val, Leu, Ile), but this percentage is the smallest (24% versus 25.4% in PsTRX*h*2, and 28.5% in PsTRX*f*). Further, one could think that the number of aromatic residues could account for the differences observed between PsTRX*h*1 (8.3%) and PsTRX*h*2 (5.4%), but it is not enough to explain the differences with PsTRX*f* (8.2%). These examples mean that not only hydrophobic core formation for alkyl-residues, or the aromatic packing must be important in attaining stability in the eukaryotic-origin family of PsTRXs, but also other factors must be taken into account to explain the differences in stability. Interestingly enough, recent theoretical calculations on several members of the TRX family, suggest that the small-scale multiple local structural variations in the residues surface are amplified into discernible global differences in the relative thermal stability [77]. Then, we examined the percentages of polar and non-polar ASA in the three modelled structures to test for differences which could explain the stability behaviour observed among the three PsTRXs, by using the VADAR website [47] (Table 5). The PsTRX*f* showed the smallest values either for the number of hydrogen-bonds or any ASA, except for the exposed non-polar ASA, which is the highest, and it is interesting to note that for PsTRX*h*1, the most stable protein, has the smallest. Thus, the non-polar ASAs is not the dominant factor in determining the stabilities among the eukaryotic-origin TRXs of pea, but rather the stability of PsTRXs is a contribution of all the ASAs involved. Thus, although the differences are not very large, the polar ΔASA among the three PsTRXs follows an opposite tendency to the stability: the more stable the protein, the smaller the polar ΔASA.

**Table 5:** Structural parameters for the PsTRXs obtained from the modelled structures a.

Structural parameter	PsTRX*h*1	PsTRX*h*2	PsTRX*f*
Number of main-chain hydrogen bonds	80	81	77
Total ASA in the folded protein	6708.6	6472.5	6291.4
Exposed non-polar ASA in the folded protein	3774.1	3950.6	3988.1
Exposed “polar and charged” ASA in the folded protein	2934.5	2521.9	2303.0
Extended non polar ASA	12360.7	12250.1	12249.8
Extended “polar and charged” ASA	7593.4	7407.6	6965.7
ΔASA non-polar (extended-folded)^b^	8586.6	8299.5	8261.7
ΔASA polar (extended-folded) b	4658.9	4885.7	4662.7

a These calculations were carried out on the VADAR web-server by using the modelled structures. The ASAs are given in Å^2^.

b These two rows were obtained from the differences of the corresponding rows in this table.

**Figure 7:**
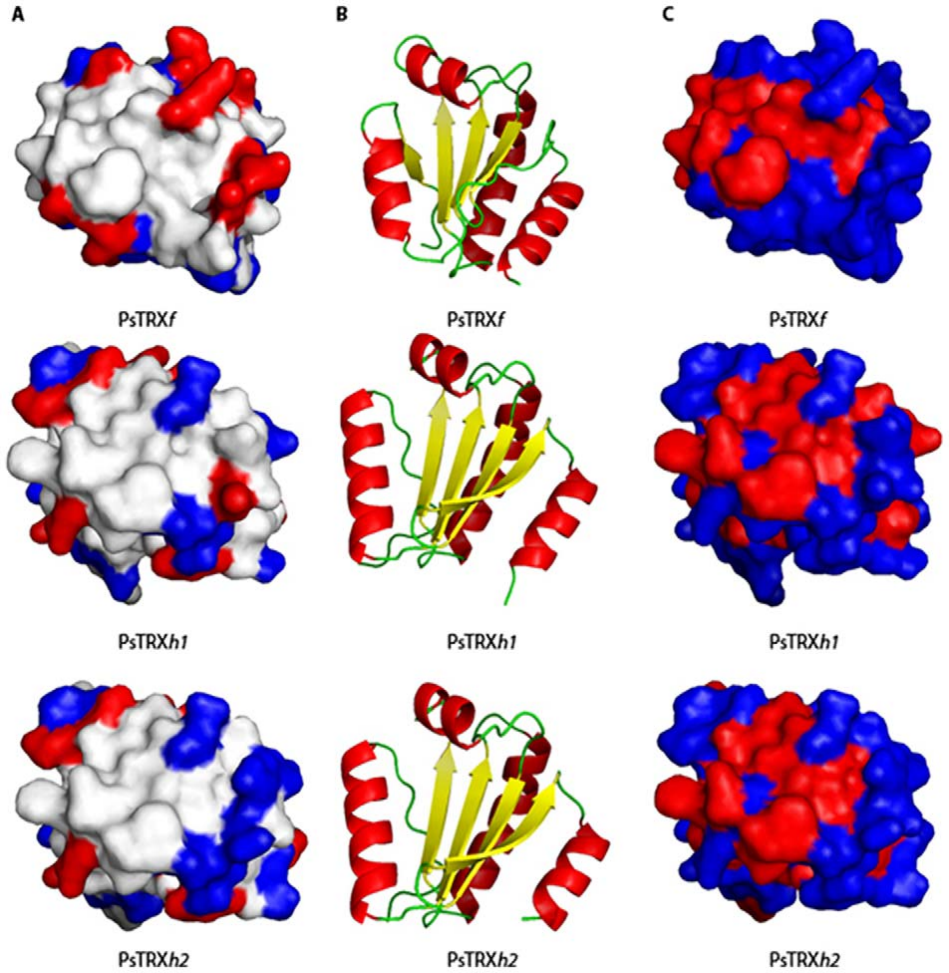
The structure of the active sites of the PsTRXs. Left column, (A), shows the charge distribution (negatively (blue) and positively (red) charged residues) in the neighbourhoods of the active site of the three TRXs; the active site of the TRXs is on the front of the structures shown. Middle column, (B), shows the ribbon representation of the scaffold of the TRXs, in the same orientation (and with a 180° turn with respect to the orientation in the left column). Right column, (C), shows the same protein orientation as (A), but showing the hydrophobicity (blue) and the hydrophilicity (red) pattern in the neighbourhood of the active site. The figures were produced with PyMOL [82].

The GdmCl-denaturations also mirrored the different thermal stabilities: the protein with the largest [GdmCl]_50%_ is PsTRX*h*1, and that with the smallest is PsTRX*h*2. But in addition, the SEC measurements suggest that the PsTRXs follow a three-state mechanism, during chemical denaturation (conversely to what is observed in thermal denaturations). We also used the ASAs to calculate the theoretically *m*-values [78]; the use of the equations provided by Pace and co-workers led to theoretical *m*-values in the range of 3.6–3.8 kcal mol^−1^ M^−1^ for the PsTRXs, which are larger than those experimentally measured (Table 4); this result is, however, not surprising since the far-UV CD spectra of the PsTRXs showed residual ellipticity, indicating the presence of structure at high [GdmCl] and/or high temperatures (data not shown). Furthermore, the three experimental *m*-values were very different among PsTRXs (Table 4), suggesting that since: (i) the folded state is highly similar (Fig. 7); and, (ii) the three *full*y unfolded states expose similar ASAs (Table 5), the final denatured states contain different amounts of residual structure in each TRX.

Finally, the protein stability data allow us to explain some aspects of the physiological behavior of these TRXs, that we have found in previous works [24], [25], [79]. We believe that the lower stability of PsTRX*f* and PsTRX*h*2 is a biochemical advantage, when it is compared with the robustness showed by PsTRX*h*1. Protein lifespan is highly determined by protein biosynthesis and degradation (turnover) according to cell requirements, and protein stability is a crucial in protein turnover. PsTRX*f* quantity and activity is finely controlled by light, since its main role is the regulation of Calvin cycle proteins. It has been shown that the expression of this gene is repressed in the dark, and the corresponding protein being degraded [79]. The lower stability of PsTRX*f* could account for a better regulation in the cell, by making easier its degradation. In a similar way, we have previously shown that PsTRX*h*2 concentration in the cell decreases when plant cells undergo oxidative stress [24]. Similarly to the PsTRX*f*, the lower chemical stability found for PsTRXh2 (comparing with PsTRX*h*1), could facilitate a faster elimination of these proteins under those conditions.

### Is there a relationship between the biophysical features and the functions of the TRXs?

Recently, it has been shown that all the eukaryotic-origin TRXs have a similar redox enzymatic mechanism (with a single-electron transfer reaction), which is different to that observed in bacterial-origin TRXs (where a nucleophilic substitution reaction also occurs) [26]. In this work, we have shown that: (i) all the eukaryotic-origin PsTRXs also show similar pH-denaturation behaviour (Fig. 3); (ii) their chemical-denaturations do not follow a two-state model; and, (iii) they do not have the same stability. Thus, the relationship between primary and tertiary structure is robust (as shown by the agreement among the three TRX modelled structures), but the link between biophysical features and primary structure is not conserved among TRXs, and it seems that there is not a direct relationship. However, we want to go a step further and to find out whether the biophysical features can be related to the different functions carried out by the three TRXs; since the biological functions of the three proteins are determined by their sequences, the finding of such relationship is challenging.

During the last 15 years we have tried to find out the physiological roles of these pea proteins. PsTRX*f* is a chloroplastiodial protein involved in the redox regulation of the photosynthetic FBasa, it is also reduced by glutathione, and it has been also localized in non-photosynthetic tissues [80]. On the other hand, the study of the citosolic forms of TRX is more complicated. The high sequence similarity between both PsTRX*h*s, together with the fact that both proteins are present in the same subcellular location, raised the question about the specific or overlapped function in cell tissues. The accumulation of negative charges around the active site of PsTRX*h*2 is larger than in PsTRX*h*1 (Fig. 7), suggesting the possibility of interactions with positively charged proteins. Conversely, the active site of PSTRX*f* has a larger accumulation of positive residues than the other two TRXs; the opposite can be said about the hydrophilic and hydrophobic pattern in the neighbourhood of their active sites (Fig. 7). Thus, based on the modelled structures, the mechanism of protein-biomolecule interactions at the active site seems to be completely different for the three proteins, and then, we hypothesize that different recognition sites should be present in the three proteins. We used the Fpocket webpage to predict the possible binding (recognition) sites in the three proteins. The program predicts four binding pockets for PsTRX*h*1, all of which are in common with PsTRX*h*2; but PsTRX*h*2 also shows an additional pocket (accounting to five predicted binding pockets), which is close to Trp15 (the residue, which we thought was responsible of the high [GdmCl]_50%_-value observed in the fluorescence); thus, we suggest that the environment around Trp15 has a local higher stability than the rest of the protein to ease protein-protein interactions (which does not mean that the flexibility of this region be lower than that of the rest of the protein).

Finally, PsTRX*f* is predicted to have nine binding pockets: all of them in common with PsTRX*h*2 and four additional ones. One of those additional binding sites is formed by the region around Cys60 (which is the cysteine responsible for the dimerization (see above), and it is the predicted interaction site with NTR [71]); furthermore, the majority of those other four additional sites involve the hydrophobic side chains of several lysine residues present in PsTRX*f* and not in the other two proteins (Fig. 1). Thus, the predicted binding sites are close to, or alternatively, are formed by, residues which we have shown to be responsible for some particular biophysical features in the TRXs: dimerization, the pH-titration or the anomalous behaviour of the fluorescence chemical-denaturation data in any of the PsTRXs. It is interesting to note that we have used the Fpocket website with the structures of TRX*h* and TRX*f* species from *Arabidopsis* to, initially, test if we could shed light on the functions of the PsTRXs; the number of predicted sites for each of the TRXs from *Arabidopsis* is different to that found for PsTRXs proteins, with four predicted binding sites for the TRX*h*1 and six binding sites for the TRX*f* (data not shown). These findings suggest that among both plant species, the protein partners recognized and the biophysical features are different.

Our previous *in vivo* [21] and *in vitro* activity measurements [24], [25], [81] showed that the three proteins are really functionally specific, interacting with different protein targets. Then, the highly conserved three-dimensional fold has allowed keeping a well-formed core, but the mutations introduced have led to: (i) different conformational stabilities; (ii) probably unlike folding kinetic pathways; and, (iii) different protein-protein pattern recognitions. Thus, subtle linkages among the inter-residue interactions restrict the possible combinations of amino acids that are able to form a well-folded structure in a family. But these subtleties are able to improve the stability in an otherwise thermo-resistant family (as PsTRX*h*1), and most importantly, the scaffold of protein interactions. This means that the sequence demands of protein-protein function are relatively rigid, with different binding pockets (some in common) for the three proteins, but the demands of structure and conformational stability *per se* (as long as a maintained core is conserved), are less so.
